# An Integrated Pharmacokinetic Study of an *Acanthopanax senticosus* Extract Preparation by Combination of Virtual Screening, Systems Pharmacology, and Multi-Component Pharmacokinetics in Rats

**DOI:** 10.3389/fphar.2020.01295

**Published:** 2020-08-14

**Authors:** Peiying Shi, Yunjiao Xie, Rongfang Xie, Zuan Lin, Hong Yao, Shuang Wu

**Affiliations:** ^1^ Department of Traditional Chinese Medicine Resource and Bee Products, College of Animal Science (College of Bee Science), Fujian Agriculture and Forestry University, Fuzhou, China; ^2^ College of Horticulture, FAFU-UCR Joint Center and Fujian Provincial Key Laboratory of Haixia Applied Plant Systems Biology, Fujian Agriculture and Forestry University, Fuzhou, China; ^3^ Department of Pharmaceutical Analysis, School of Pharmacy, Fujian Medical University, Fuzhou, China

**Keywords:** integrated pharmacokinetics, *Acanthopanax senticosus* extract preparation, multi-component pharmacokinetics, systems pharmacology, virtual screening

## Abstract

In this paper, the integrated pharmacokinetics (PK) of an *Acanthopanax senticosus* extract preparation (ASEP, named as Ciwujia injection in clinic in China) was explored by combining with multi-component PK in rats, virtual screening, systems pharmacology and molecular docking. Firstly, the ingredients in ASEP with high contents and detectable property in rat plasma were selected. Next, the PK study of the resulted ingredients was performed in rats (1.76 ml/kg and 3.52 ml/kg of 5 times concentrated ASEP, single i.v.). Meanwhile, the drug targets for the ingredients screened out were predicted by using a target fishing online server, PharmMapper (http://www.lilab-ecust.cn/pharmmapper/) with a fit filtration threshold of z’-score >0. Next, the network pharmacology, molecular docking, diseases ontology (DO) analysis, and Kyoto encyclopedia of genes and genomes (KEGG) pathway enrichment analysis were performed respectively for the predicted targets. Finally, the supporting evidences were obtained to characterize the PK markers and carry out the integrated PK study with “plasma-drug concentration sum” or “plasma-drug AUC weighted” methods. As a result, 6 ingredients, involving 5-caffeoylquinic acid (5-CQA), 3-CQA, 4-CQA, protocatechuic acid, eleutheroside B, and gentiopicroside were selected, and their PK profiles were elucidated. The 6 ingredients were highly related to arteriosclerotic cardiovascular disease and atherosclerosis and could mainly interact with similar targets, e.g., GSK3B, PDPK1, PLAU, etc., or pathways, e.g., Insulin, VEGF, FoxO, etc, providing the basis for integrating plasma drug concentration. Ultimately, the 6 ingredients were considered as PK markers and the whole *in vivo* process of ASEP were characterized. Our study would enhance understanding of the therapeutic effects and mechanisms of ASEP against cardiovascular diseases, and provided useful insights for future integrated PK study on anti-cardiovascular diseases TCM injections.

## Introduction

*Acanthopanax senticosus* (Rupr. et Maxim) Harms, also called Ciwujia in China, belonging to the family of *Araliaceae*, is a hardy shrub, native to the northeastern region of China, Korea, and Japan, and the far-eastern region of Russia (Huang et al., 2011). This shrub has been utilized for the treatment of various ailments such as cancer (Huang et al., 2005), cardiovascular diseases (CVDs) (Li, 2003), cerebrovascular diseases (Li et al., 2009), neurodegenerative diseases (Shi and Chen, 2017), diabetes (Yu et al., 2016), and so on. An *Acanthopanax senticosus* extract preparation (ASEP, named as Ciwujia injection in clinic in China) is the sterile aqueous solution made by extracting and processing the root or rhizome of *A. senticosus* (Ciwujia Zhusheye quality standard (WS3-B-3425-98), 1998). Its main components, including lignans, phenolic acids, nucleosides, coumarins, and terpenoids, etc., have been identified using high performance liquid chromatography combined with quadrupole time-of-flight mass spectrometry (Huang et al., 2014; Xie et al., 2019). ASEP has been widely used to treat cardiovascular and cerebrovascular diseases, such as coronary heart disease, angina pectoris (Li, 2004; Lei, 2015), cerebral infarction (Qin et al., 2020), cerebral hemorrhage (Fan, 2016), and so on (Fan et al., 2003). However, as one of the frequently used traditional Chinese medicine (TCM) injection in clinic, ASEP’s adverse drug reactions (ADRs) have also been reported. The ADR cases were mainly general reactions (allergic shock), skin lesions (urticaria), and respiratory system reactions (dyspnea) (Hu et al., 2010). Clinical misuse could be one of the factors relating to the ADRs induced by TCM injections (Zhang et al., 2015). A recent report on 1,270 cases of ADRs induced by ASEP concluded that attention should be paid to safe and rational use of ASEP, and clinical nursing and monitoring of ADRs had to be enhanced to prevent over-indications and over-dosage medication (Wen et al., 2020). Thus, formulating the rational dosage regimens through understanding the *in vivo* pharmacokinetics (PK) of TCM injections is of great significance.

Previously, PK studies for ASEP were mainly referring to two or few ingredients in the preparation. For examples, an HPLC method was developed for the PK study of two lignans, eleutheroside B (EB, also referred as syringin) and eleutheroside E in rat plasma and tissue following administration of ASEP (Feng et al., 2006). Besides, a rapid and effective liquid chromatography tandem mass spectrometry method was developed to determine the above two lignans and isofraxidin, in rat plasma and applied to the PK study after intravenous administration of each monomer and ASEP (Fan et al., 2014). Using the same method, the PK profiles of ASEP in normal rats and cerebral ischemia-reperfusion rats were investigated, respectively, and the results showed that compared with the normal group, syringin accumulated in the cerebral ischemia-reperfusion rats, as well as the elimination of isofraxidin changed (Fan et al., 2015). These studies have brought about valuable information for understanding the *in vivo* PK of ASEP. However, many other main ingredients in ASEP, including 3-caffeoylquinic acid (3-CQA), 4-CQA, 5-CQA, protocatechuic acid (PCA), and gentiopicroside (GPS) (Xie et al., 2019), had little attention in PK study of ASEP.

In addition, the integrated PK study of a TCM means to integrate the blood drug concentration-time profiles of all PK markers using modeling methods to obtain one set of PK parameters to represent the whole *in vivo* process of a TCM. It is based on the multi-components PK, as well as takes into account the pharmacological effects and the contribution weights of the multi-components to the treatment effects. Thus, the integrated PK parameters could be helpful for the dosage regimen design of complex TCMs’ preparations (Yao et al., 2017; Shi et al., 2018; Yao et al., 2018; Huang et al., 2019). In the evaluation of efficacy, besides *in vivo* and *in vitro* experiments, systems pharmacology has received more and more attention due to its serviceability on predicting the therapeutic effects and mechanisms of multiple ingredients in TCMs (Fu et al., 2014; Liu et al., 2016; Zhang et al., 2016). Furthermore, systems pharmacology has been considered in predicting the main effect substances and identifying pharmacokinetic markers for a TCM, which was helpful to the integrated PK study of the TCM (Yao et al., 2018). Up to date, the systems pharmacology study on ASEP against CVDs has not been reported.

In this paper, an integrated PK study of ASEP in rats was carried out by the combination of multi-component PK, virtual screening, systems pharmacology and bioinformatics analysis. The PK study of multiple ingredients from ASEP in rats identified the main ingredients with favorable PK properties. The network pharmacology, molecular docking and diseases ontology (DO) analysis, and Kyoto encyclopedia of genes and genomes (KEGG) pathway enrichment analysis were further carried out to reveal the common pathways of the main active ingredients in ASEP against CVDs, which provided evidences to support the integration of the plasma drug concentration of multiple ingredients for the integrated PK study of ASEP.

## Materials and Methods

### Chemicals and Reagents

ASEPs were offered by Heilongjiang Duoduo Pharmaceutical Co., Ltd. (batch no. 17060129). The contents of main ingredients in ASEP were qauntified by HPLC-UV, including 978.62 μg/ml of 5-CQA, 666 μg/ml of PCA, 528 μg/ml of 3-CQA, 953 μg/ml of EB, 721 μg/ml of 4-CQA, and 128.5 μg/ml of GPS.

Reference compounds (HPLC purity, >98%), referring to PCA, 3-CQA, 4-CQA, 5-CQA, EB, GPS and bergenin [internal standard (IS)] were obtained from Shanghai Ronghe Medicine Technology Development Co., Ltd.

HPLC-grade methanol was from Sigma (USA). Water was prepared by the Kertone Mini D system (Kertone, Changsha, China). Formic acid was obtained from aladdin Chemistry Co., Ltd. (Shanghai, China).

### Experimental Animals

The animal experiments were conducted in line with the Guide for the Care and Use of Laboratory Animals (published by the USA National Institutes of Health, NIH Publication no. 85-23, revised 1996), which were approved by the Animal Care and Use Committee of the College of Animal Science (College of Bee Science), Fujian Agriculture and Forestry University (Approval Number: PZCASFAFU2019004). Twelve seven-week-old male Sprague-Dawley rats with bodyweight of 200 ± 20 g were provided from Laboratory Animal Center in Fujian Medical University (Fuzhou, China). They were housed in rat cages (48 × 29 × 18 cm^3^), in a unidirectional airflow room with controlled temperature (22 ± 2 °C), relative humidity (40%–70%), and a 12 h light/dark cycle. The animals were able to freely take commercial food and filtered tap water, and fasted with free access to water for 12 h before administration. All rats were acclimated to the facilities and environment for 7 days before the experiments.

### Instrument and Analytical Conditions

An LC-MS system (LC-MS 8040, Shimadzu, Japan) with an Ultimate^®^XB-C_18_ column (4.6×50 mm, 3.5 μm) was used in the experiments. The gradient elution was carried out by the mobile phase water with 0.1% formic acid (A) and methanol (B). The gradient program was as follows: 0–8 min, start with 20% B, then linearly increase to 40% B; 8-10 min, linearly increase to 75% B; 10–10.1 min, linearly increase to 95% B and kept at 95% B from 10.1 min to 14 min. The column temperature and flow rate were at 35 °C and 0.5 ml/min, resepectively. 5 μl sample solution was injected for the HPLC-MS analysis. The electrospray ionization (ESI) source conditions were as follows: block heating temperature was kept at 400 °C; desolvation line temperature was set at 250 °C; dry gas (nitrogen) flow rate was kept at 15 L/min; and auxiliary gas (nitrogen) flow rate was 3 L/min. Quantification was performed using selective ion monitoring (SIM) in negative ionization mode by monitoring the [M-H]^-^ ions at *m/z* 353 for 5-CQA, 3-CQA and 4-CQA, *m/z* 153 for PCA, *m/z* 327 for IS, as well as [M+HCOO]^-^ ions at *m/z* 417 for EB and *m/z* 401 for GPS.

### PK Experiment

Rats were randomly divided into two groups (*n*=3). 5 times concentrated ASEP (4,893.1 μg/ml of 5-CQA, 3,330 μg/ml of PCA, 2,640 μg/ml of 3-CQA, 4,765 μg/ml of EB, 3,605 μg/ml of 4-CQA, and 642.5 μg/ml of GPS) was intravenously injected through the tail vein (1.76 ml/kg and 3.52 ml/kg dosage, respectively). Each rat was fixed in a rat fixator without local anesthetic when the concentrated ASEP was injected. Blood was taken from the cut-tail before administration and at 2.5, 5, 10, 15, 20, 30, 45 min, and 1, 1.5, 2, 3, 4, 6, 8, 12, and 24 h following intravenous injection. 2 ml of normal saline was administrated to each rat by intraperitoneal injection after 30 min sampling. The plasma samples were separated by centrifugation, followed by storing at -20 °C before analysis.

### Plasma Sample Preparation

100 μl of plasma was mixed with 10 μl of IS solution (10 μg/ml of bergenin) and 300 μl of methanol, followed by vortexing for 3 min and centrifuging for 10 min (at 13,000 rpm and 4 °C). 5 μl of the resulting supernatant was analyzed by LC-MS.

### Method Validation

According to the “Guidelines for non-clinical pharmacokinetics of drugs” issued by Centre for Drug Evaluation, National Medical Products Administration, China (Guidelines for non-clinical pharmacokinetics of drugs, 2014), the LC-MS method was validated by linearity, limits of detection (LOD), limits of quantification (LOQ), precision, accuracy, extraction recovery, matrix effect and stability of the 6 compounds in rat plasma. In brief, blank rat plasma was obtained from the other six rats. The standard mixture solution containing the 6 compounds (with the concentration of 1 mg/ml for each) was diluted with menthol to prepare a series of working solution containing the 6 compounds (concentration level: 20, 50, 100, 250, 500 ng/ml and 1, 2.5, 5, 10, 25, 50, 100, 125 150, 200 μg/ml) and IS (2.5μg/ml). For calibration curve, 10 μl of the series of working solution containing the IS (2.5μg/ml) was added into 100 μl of blank plasma. Finally, 15 data points (2, 5, 10, 25, 50, 100, 250, 500 ng/ml and 1, 2.5, 5, 10, 12.5 15, 20 μg/ml) were used in the calibration curve linearity. Calibration curves were plotted by weighted linear regression of the peak area ratio of analyte to the IS against the related nominal concentration of the analyte. LOD and LOQ were defined as the detectable concentration at which the ratio of signal to noise was about 3 and 10, respectively (Huang et al., 2019). Intra- and inter-day accuracy and precision were evaluated by analyzing spiked rat plasma samples (QC samples, n=5) at three concentration levels on one or three validation days, respectively, and the results were presented as relative error (RE) and relative standard deviation (RSD), respectively (Huang et al., 2019). The extraction recovery and matrix effect were also assessed at the above-mentioned three concentration levels for the 6 ingredients in the spiked plasma samples (Huang et al., 2019). Stability was examined under the condition of setting un-treated plasma samples at ambient temperature for 6 h, and extracted plasma samples in auto-sampler for 10 h, as well as setting plasma samples for three freeze-thaw cycles and at −80°C for 15 days (Guidelines for non-clinical pharmacokinetics of drugs, 2014).

### Data Processing

A DAS 3.0 software (Chinese Pharmacologic Society, Beijing, China) was used to calculate the noncompartmental PK parameters. All the results were presented as mean ± SD, and statistics analysis was done with a single-tailed Student’s t test in the work.

### Target Finishing

Firstly, the structure of the ingredients was built, followed by energy minimization and saved as mol2 type files with molecular docking software Sybyl-X (version 1.3, TRIPOS Inc.). A target fishing web-server (PharmMapper Server, available online at http://www.lilab-ecust.cn/pharmmapper/on April 17, 2020) was used to seek for the potential target proteins for each ingredient with the mol2 type file and pharmacophore mapping method (Liu et al., 2010; Wang et al., 2016; Wang et al., 2017). Subsequently, top 300 targets were predicted from the PharmMapper. Among the targets, those with z’-score >0 and definite CVDs relativity were selected as the potential targets for the correlative ingredients.

### Compound-Target Network Construction

Next, the gene symbols of potential target proteins were obtained by searching the Uniprot databases (http://www.uniprot.org/uniprot/), followed by sending the gene symbol to the molecule annotation systems (MAS 3, http://bioinfo.capitalbio.com/mas3/; or David 6.8, https://david.ncifcrf.gov/summary.jsp) to inquire about the possible CVDs. The genes with the CVDs relativity were selected to build the C–T network with Cytoscape (version 3.6.0, http://www.cytoscape.org/). The resulting network consisted of node and edge. Nodes referred to the molecules involving ingredients and targets, while edges showed the connections between nodes. The connection degree was defined as the edge numbers of one node connected with others nodes.

### Molecular Docking

The two docking softwares, SYBYL-X 1.3 and LeDock (http://www.lephar.com/software.htm) were used as confirmatory tool for the studied ingredients and their potential targets. The Surflex-docking module in SYBYL is based on a “ProtoMol”, a representative protein activity docking pocket, which can be automatically and/or user-defined generated. Before initiating the docking simulations, the co-crystallized ligand and structural water molecules were removed from the crystal structure and the polar hydrogen atoms were added in SYBYL software. LeDock is an easily-used molecular docking program, which use “PDB” protein file and “MOL2” file as acceptor and ligand, respectively to model protein-compound reaction and calculate the binding energy (the binding energy < 0 kcal/mol is predicted as favorable for binding reaction). The protein-ligand interactions were viewed by PyMOL.

### Bioinformatics

Disease Ontology database was utilized to characterize the enriched diseases by a two-tailed Fisher’s exact test through testing the enrichment of the target protein against all identified proteins. The diseases with a corrected p-value < 0.01 were considered significant. The results were visualized by a dot plot using the “dotplot” function in R-package. KEGG database was applied to characterize enriched pathways by a two-tailed Fisher’s exact test *via* testing the enrichment of the targets against all potential target proteins. The pathways with a corrected p-value < 0.05 were considered significant. The results were visualized by a bar plot using the “barplot” function in the R-package.

For further pathway hierarchical clustering based on the predicted target proteins, we first collected all the results of each ingredient obtained after KEGG pathway enrichment along with their P values <0.05 and integrate them into one dataset, and then this filtered P value matrix was transformed by the function x = −log10 (P value). Finally, these x values were z-transformed for each functional category. These z scores were then clustered by one-way hierarchical clustering (Euclidean distance, average linkage clustering) in Genesis. Cluster membership were visualized by a heat map using the “pheatmap” function from the R-package.

## Results and Discussion

### Multiple Component PK Study of ASEP in Rats

In the preliminary examination, 13 of the 22 ingredients in the preparation can be detected at the PK time point of 5 min beyond 0.5 ng/ml in rats’ plasma after single *i.v.* administration of 3.52 ml/kg of 5 times concentrated ASEP. However, beyond 5 min, only the 6 ingredients, including 5-CQA, 3-CQA, 4-CQA, PCA, EB, and GPS possessed the detectable property in rat plasma (each ingredient concentration was at least beyond 0.5 ng/ml at the PK time points of 2.5, 5, 10, 15, and 20 min), suggesting their favorable PK properties possibly due to their high contents in the preparation. Subsequently, only the 6 ingredients, including 5-CQA, 3-CQA, 4-CQA, PCA, EB, and GPS were chosen for PK study.

**Figure 1:**
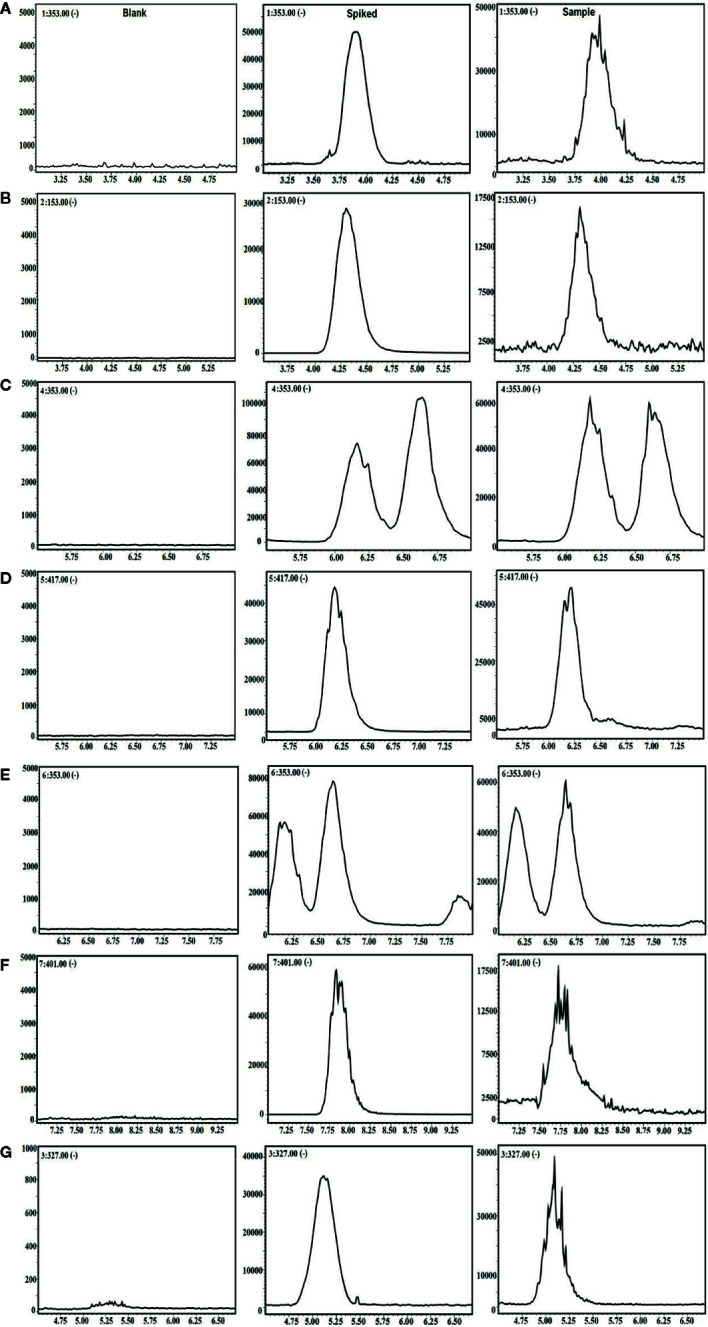
The selective ion monitoring (SIM) chromatograms of 6 compounds and internal standard (IS) in negative mode. **(A)** 5-CQA; **(B)** PCA; **(C)** 3-CQA; **(D)** EB; **(E)** 4-CQA; **(F)** GPS; and **(G)** IS.

For further LC-MS bioanalysis, the method was validated including system linearity, precision, accuracy, stability, etc. for the 6 ingredients in plasma. As shown in **Figure 1**, the typical SIM chromatograms for the 6 ingredients in blank, spiked, and drug plasma after intravenous administration for 5 min demonstrate no interference could be observed, suggesting the good selectivity of the presented bioanalysis method.

As listed in **Supplementary Table S1**, calibration curves of the 6 ingredients in rat plasma possess good linearity with high correlation coefficients (r^2^ > 0.990) within the tested ranges. **Supplementary Table S2** lists the results of intra- and inter-day precision and accuracy for the determination of 6 ingredients in QC samples. For all the 6 compounds, the intra- and inter-day precision values (RSDs) were 1.41%–14.94% and 2.05–14.5%, respectively. The intra-day RE values for precisions were -10.19–2.2% (except the value of 17.2% for the low concentration of QC (LQC) samples of 4-CQA), and those of inter-day precisions were -8.5%–12.14%. All these results indicated that the presented method was accurate and reliable. As listed in **Supplementary Table S3**, the extraction recoveries of the 6 ingredients were within the range of 81.51%–113.55% with RSD values of 2.4%–12.47%, and the matrix effects were in the range of 72.08%–128.61% with RSD values of 1.43%–9.92%, which suggested that the presented method was reliable for the determination of the 6 ingredients in rat plasma. **Supplementary Table S4** lists the test results of the sample stability. It indicated that the accuracy (expressed as RE) were in a range from −14.91% to 14.43% for MQC and HQC samples and from −16.89% to 19.9% for LQC samples, suggesting the analytes stable at the tested condition.

**Figure 2:**
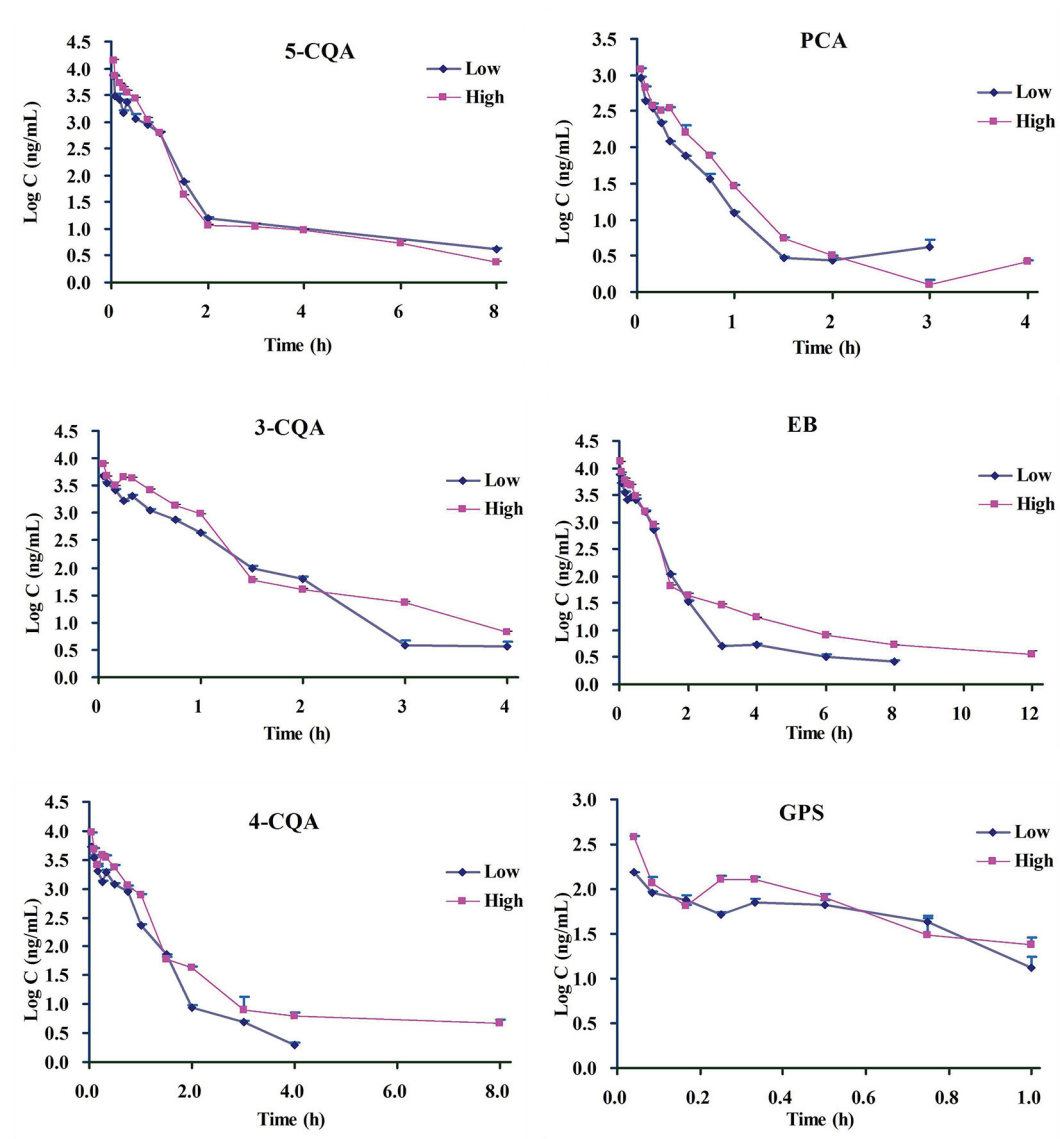
The mean drug plasma concentration–time curves for the 6 ingredients after *i.v.* administration of *Acanthopanax senticosus* extract preparation (ASEP) (low and high dosages) (Mean ± SD, *n*=3).

According to the validation results, it can conclude that the presented LC-MS method could meet with requirement for the simultaneous determination of the 6 ingredients in rat plasma. By using this LC-MS method, the plasma drug concentrations of the 6 ingredients in rats were determined after administration (1.76 ml/kg and 3.52 ml/kg of 5 times concentrated ASEP, single *i.v.*). The plasma drug concentration-time curves and PK parameters for the 6 ingredients are shown in **Figure 2** and **Table 1**, respectively. It showed that 3-CQA, 4-CQA, PCA, 5-CQA, and GPS were eliminated rapidly (0.23 h ≤ *t*_1/2_ ≤ 2.01 h), while EB was eliminated relatively slowly (*t*_1/2_ ≥ 3.17 h) in rat blood. Meanwhile, the AUC values ordering for these ingredients is EB > 5-CQA > 3-CQA > 4-CQA > PCA > GPS.

**Table 1:** The pharmacokinetics (PK) parameters of the six ingredients in rat plasma after i.v. administration of *Acanthopanax senticosus* extract preparation (ASEP) (Mean ± SD, n=3).

Analyte	Group	*t*_1/2_	AUC_0-t_	AUC_0-∞_	Vd	Cl	MRT_0-t_	MRT_0-∞_
		(h)	(h·ng/ml)	(h·ng/ml)	(L)	(L/h)	(h)	(h)
5-CQA	Low	0.84 ± 0.00	2,244.68 ± 109.65	2,249.80 ± 109.69	0.93 ± 0.05	0.77 ± 0.04	0.44 ± 0.02	0.46 ± 0.02
	High	2.01 ± 0.15	4,006.71 ± 42.76	4,013.66 ± 42.67	2.49 ± 0.18	0.86 ± 0.01	0.33 ± 0.00	0.35 ± 0.00
PCA	Low	0.35 ± 0.01	201.55 ± 4.82	203.70 ± 5.08	2.91 ± 0.07	5.76 ± 0.14	0.25 ± 0.01	0.28 ± 0.02
	High	0.40 ± 0.00	308.40 ± 14.20	309.91 ± 14.10	4.39 ± 0.22	7.57 ± 0.34	0.30 ± 0.01	0.32 ± 0.01
3-CQA	Low	0.36 ± 0.01	1,826.14 ± 37.87	1,828.07 ± 37.96	0.26 ± 0.01	0.51 ± 0.01	0.43 ± 0.02	0.43 ± 0.02
	High	0.81 ± 0.00	3,411.11 ± 37.37	3,418.92 ± 37.87	0.64 ± 0.00	0.54 ± 0.01	0.42 ± 0.00	0.43 ± 0.00
EB	Low	4.35 ± 0.10	3,041.09 ± 31.03	3,057.81 ± 31.55	3.45 ± 0.10	0.55 ± 0.01	0.44 ± 0.00	0.52 ± 0.01
	High	3.17 ± 1.09	4,528.57 ± 58.27	4,544.97 ± 58.20	3.38 ± 1.15	0.74 ± 0.01	0.44 ± 0.01	0.50 ± 0.02
4-CQA	Low	0.93 ± 0.08	1,747.89 ± 64.52	1,750.59 ± 64.24	0.98 ± 0.11	0.73 ± 0.03	0.36 ± 0.00	0.37 ± 0.00
	High	0.66 ± 0.01	3,156.93 ± 68.74	3,161.48 ± 69.18	0.76 ± 0.02	0.80 ± 0.02	0.42 ± 0.01	0.43 ± 0.01
GPS	Low	0.23 ± 0.06	63.57 ± 2.53	68.23 ± 1.88	1.08 ± 0.26	3.32 ± 0.09	0.36 ± 0.01	0.42 ± 0.05
	High	0.26 ± 0.04	108.95 ± 5.92	118.37 ± 6.75	1.45 ± 0.16	3.83 ± 0.23	0.26 ± 0.01	0.35 ± 0.04
Con.sum	Low	0.58 ± 0.00	8,977.67 ± 210.66	8,983.44 ± 210.61	0.63 ± 0.02	0.75 ± 0.02	0.41 ± 0.01	0.42 ± 0.01
	High	2.01 ± 0.23	15,408.36 ± 127.56	15,419.09 ± 125.36	2.54 ± 0.30	0.88 ± 0.01	0.41 ± 0.00	0.42 ± 0.00
AUC integrated	Low	0.59 ± 0.00	2,265.82 ± 49.05	2,267.48 ± 49.04	0.55 ± 0.01	0.64 ± 0.01	0.42 ± 0.01	0.42 ± 0.01
	High	2.55 ± 0.68	3,778.67 ± 33.98	3,782.71 ± 32.53	2.78 ± 0.76	0.76 ± 0.01	0.41 ± 0.00	0.42 ± 0.01

### Virtual Screening

The drug similarity (DS) of 22 ingredients identified in ASEP in our previous study (Xie et al., 2019) was predicted through DrugBank database (http://www.drugbank.ca/), and 8 ingredients, including PCA (DS = 1.00), 3-CQA (DS = 1.00), Caffiec Acid (DS = 1.00), EB (DS = 0.92), 5-CQA (DS = 0.82), GPS (DS = 0.72), isofraxidin (DS = 0.70), and 4-CQA (DS = 0.65) were selected based on DS≥0.6. These results combined with the PK profiles and contents of the ingredients in ASEP synergistically suggested that only the six compounds, including PCA, 3-CQA, EB, 5-CQA, 4-CQA, and GPS, could be reasonably considered as the main therapeutic factors against CVDs *in vivo*. Thus, the six compounds were further examined for target fishing.

**Figure 3:**
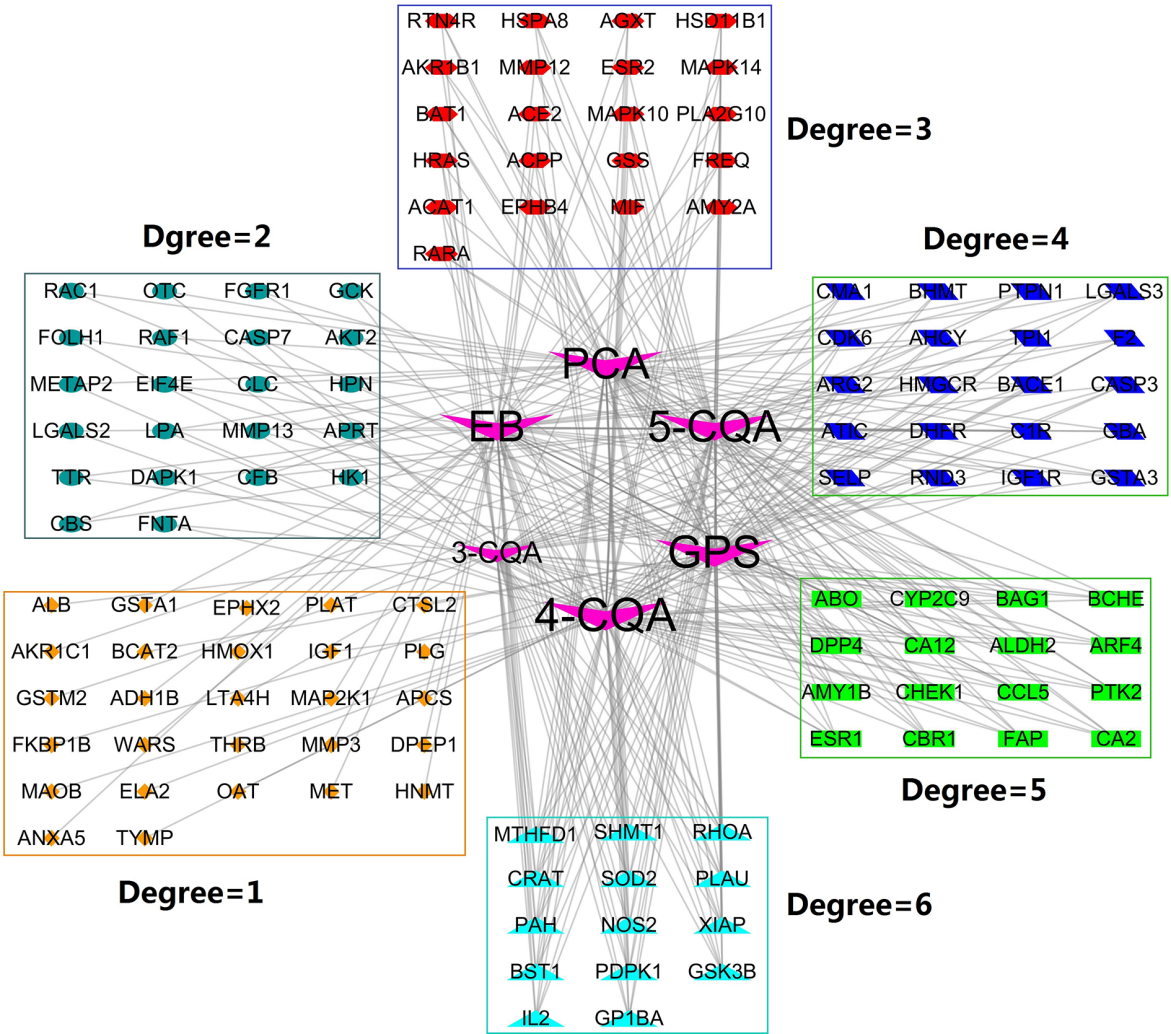
Compound-Target (C-T) network. V shape nodes represent compounds, which go counterclockwise from 4-CQA to 3-CQA according to their degree size. The other shapes represent the potential targets and clustered in rectangles according to their degree values.

Using the on-line PharmMapper Server, a total of 120 proteins (**Figure 3**) with the cardiovascular and cerebrovascular affairs relevance (e.g., ischemia, arteriosclerosis, angina, arteriosclerosis, Parkinson’s disease, shock, hypercholesterolemia, hyperlipidemia, infarction, Alzheimer’s disease, oxidative injury, hypertrophy, and thrombotic diseases) (**Figure 5**) were predicted as the targets.

### Compound-Target Network

To probe the possible interactions between compounds and proteins, we constructed the compound-target network (**Figure 3**) by mapping the six compounds to the predicted 120 targets. The obtained network consisted of 126 nodes and 312 edges, in which the node 4-CQA owned the highest connection degree (70) and the next nodes were GPS (degree = 67), 5-CQA (degree = 66), EB (degree = 65), PCA (degree = 65), and 3-CQA (degree = 45). The results suggested that 4-CQA, GPS, 5-CQA, EB, and PCA could play an important role in exerting cardiovascular or cerebrovascular effects for ASEP. Meanwhile, 14 targets, including GSK3B, PDPK1, SOD2, PAH, NOS2, XIAP, SHMT1, RHOA, PLAU, MTHFD1, IL2, GP1BA, CRAT, and BST1 corresponded to the six studied ingredients, suggesting that these proteins could be the main targets of ASEP’s multi-ingredients *in vivo*. In addition, the percentage of nodes with a degree ≥3 is 59.17%, indicating that the six ingredients shared many targets.

### Molecular Docking

**Figure 4:**
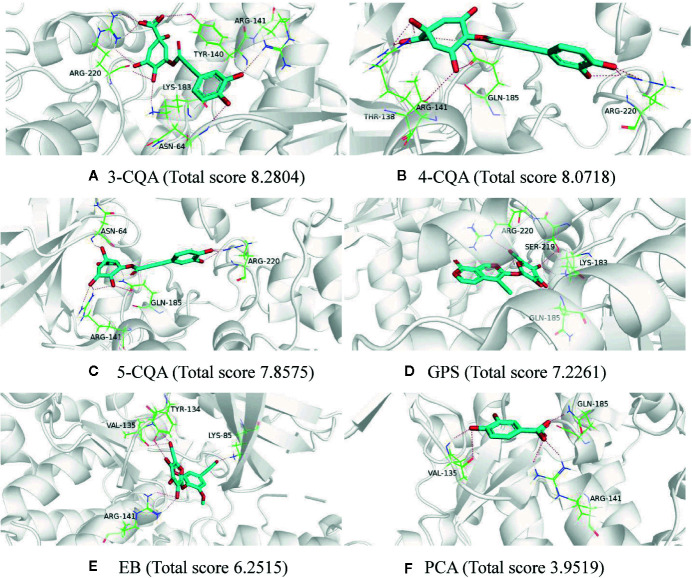
Molecular docking charts of 6 compounds (including 3-CQA **(A)**, 4-CQA **(B)**, 5-CQA **(C)**, GPS **(D)**, EB **(E)** and PCA **(F)**) and GSK3B (PDB ID 1q4l).

To further validate the potential targets possessing good affinity to the six studied ingredients, molecular docking was performed using the software SYBYL-X 1.3 for these ingredients with the 14 high relevance degree proteins, including GSK3B, PDPK1, SOD2, PAH, NOS2, XIAP, SHMT1, RHOA, PLAU, MTHFD1, IL2, GP1BA, CRAT, and BST1. As shown in **Supplementary Table S5**, all the six ingredients could dock stably with the 14 mentioned proteins, and almost each protein could interact well (docking total score values >5) with more than one ingredient. Taking the protein GSK3B (PDB ID 1q4l) as an example, all the six ingredients could fit with it (**Figure 4**). 3-CQA could form hydrogen bonds with the amino acids residues ASN-64, TYR-140, ARG-141, LYS-183, and ARG-220 and it could fit well with the activity cavity of GSK3B with a highest fit total score value (8.2804). Except for PCA (docking total score 3.9519), the rest four ingredients, 4-CQA, 5-CQA, GPS and EB could also interact well with the activity package of GSK3B (the docking total score values were 8.0718, 7.8575, 7.2261, and 6.2515, respectively). Meanwhile, to further support the virtual screening results, another molecular docking software LeDock (http://www.lephar.com/software.htm) was complementarily used to model and calculate the binding energy between the potential targets and 6 ingredients in ASEP. The results are listed as **Supplementary Table S6**. As shown in the results, all the studied ingredients have favorable binding energy (<0 kcal/mol) with their relative potential target proteins, which adds chips to the reliability of the virtual screening results. These suggested that the potential 120 target proteins found from target fishing could indeed possess affinity in certain extent.

### Bioinformatics Analysis

**Figure 5:**
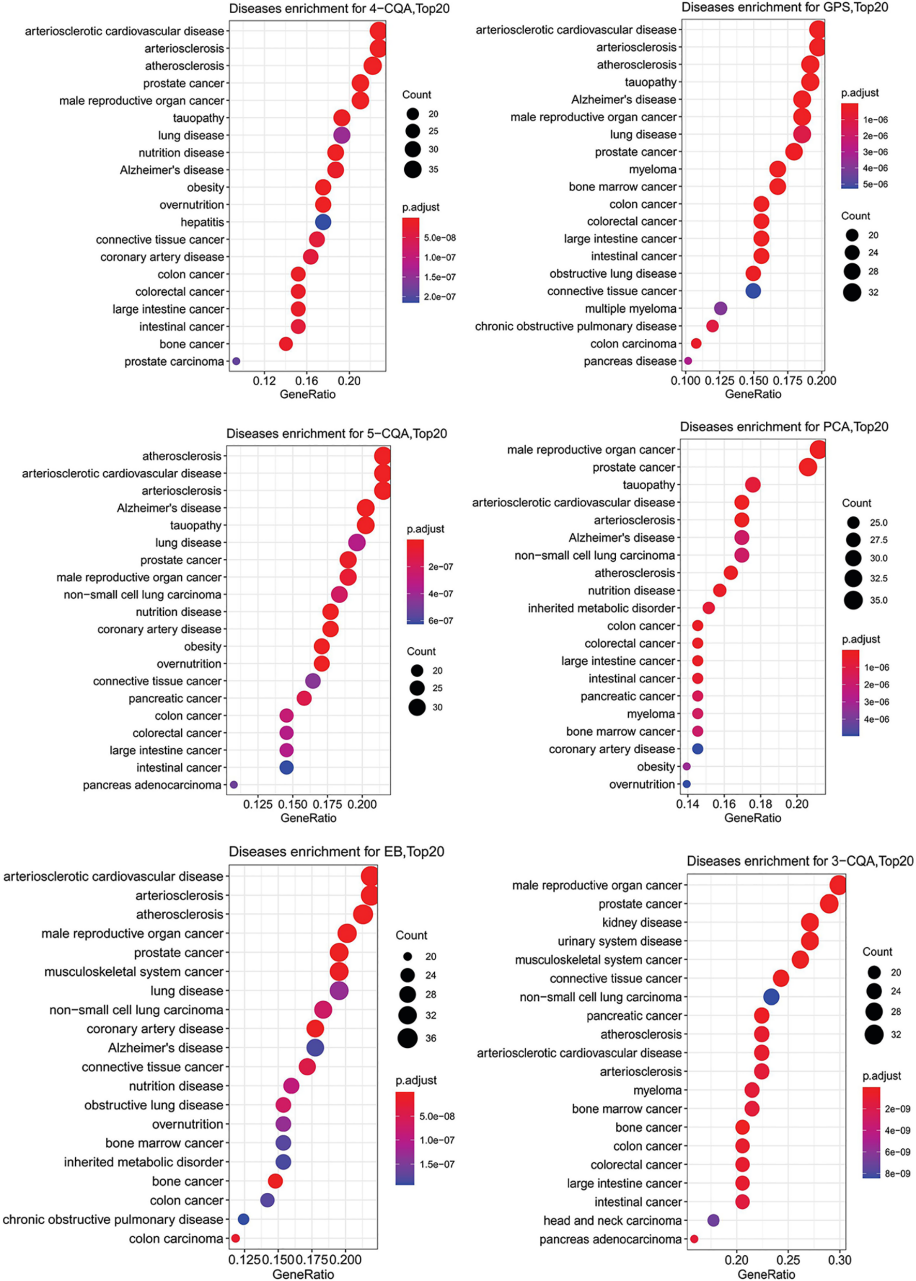
Dotplots of diseases ontology (DO) enrichment analysis (Top20 results) for the six ingredients, 4-CQA, gentiopicroside (GPS), 5-CQA, protocatechuic acid (PCA), eleutheroside B (EB), and 3-CQA.

To further study the relationship of the ingredients and the targets predicted with cardiovascular or cerebrovascular affairs, DO enrichment analysis was performed for each ingredient by R-package with the DO database. As shown in **Figure 5**, although the two ingredients, PCA and 3-CQA had the best matching degree to male reproductive cancer and prostate cancer, all the six ingredients studied showed similar and high relevance to the CVDs, including arteriosclerotic cardiovascular disease, arteriosclerosis and atherosclerosis. These results suggested that all the six ingredients could be the main active substances against CVDs in ASEP.

**Figure 6:**
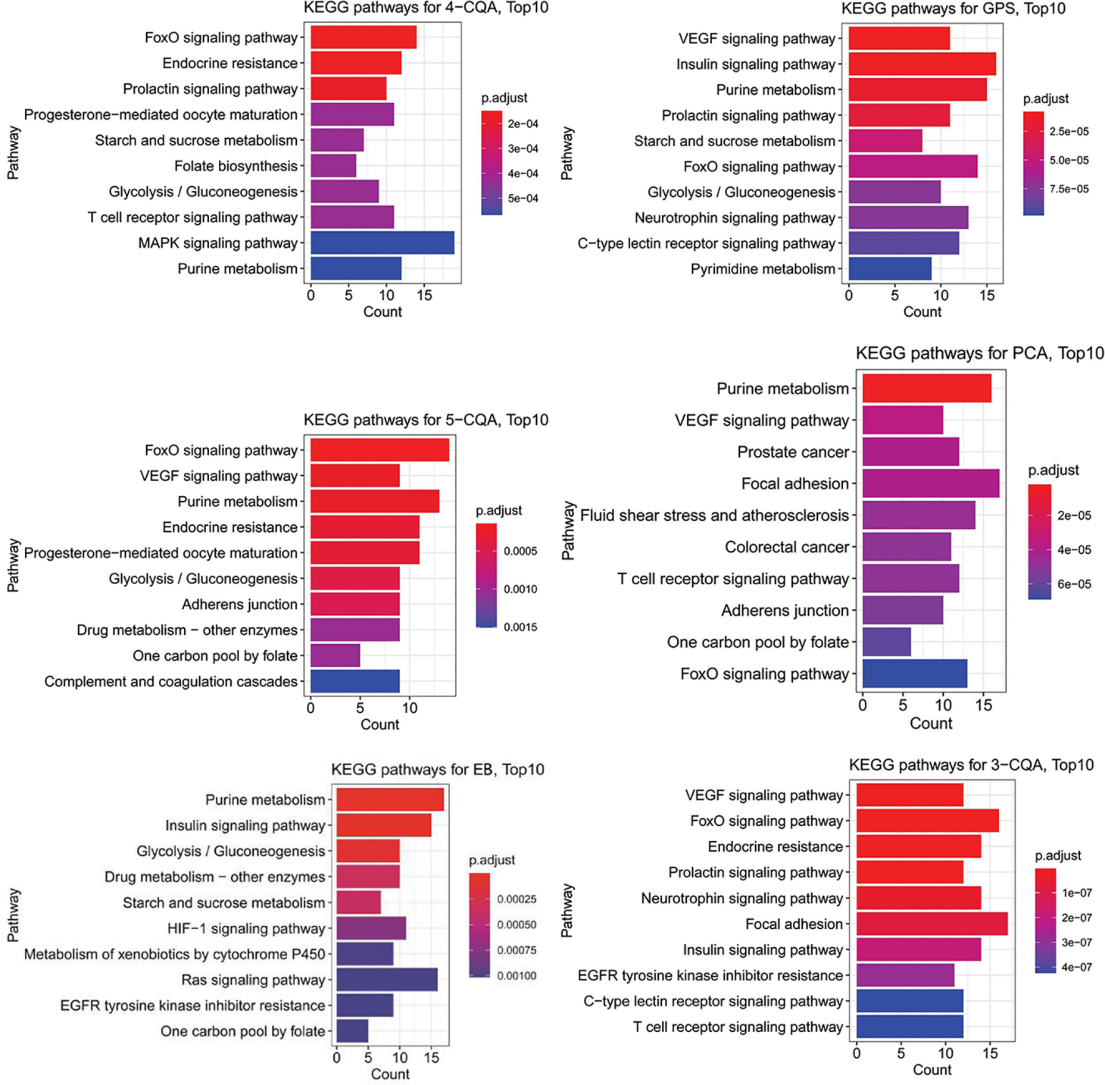
Barplots of KEGG pathway enrichment analysis (top10 results) for the six main ingredients, 4-CQA, gentiopicroside (GPS), 5-CQA, protocatechuic acid (PCA), eleutheroside B (EB), and 3-CQA in *Acanthopanax senticosus* extract preparation (ASEP).

**Figure 7:**
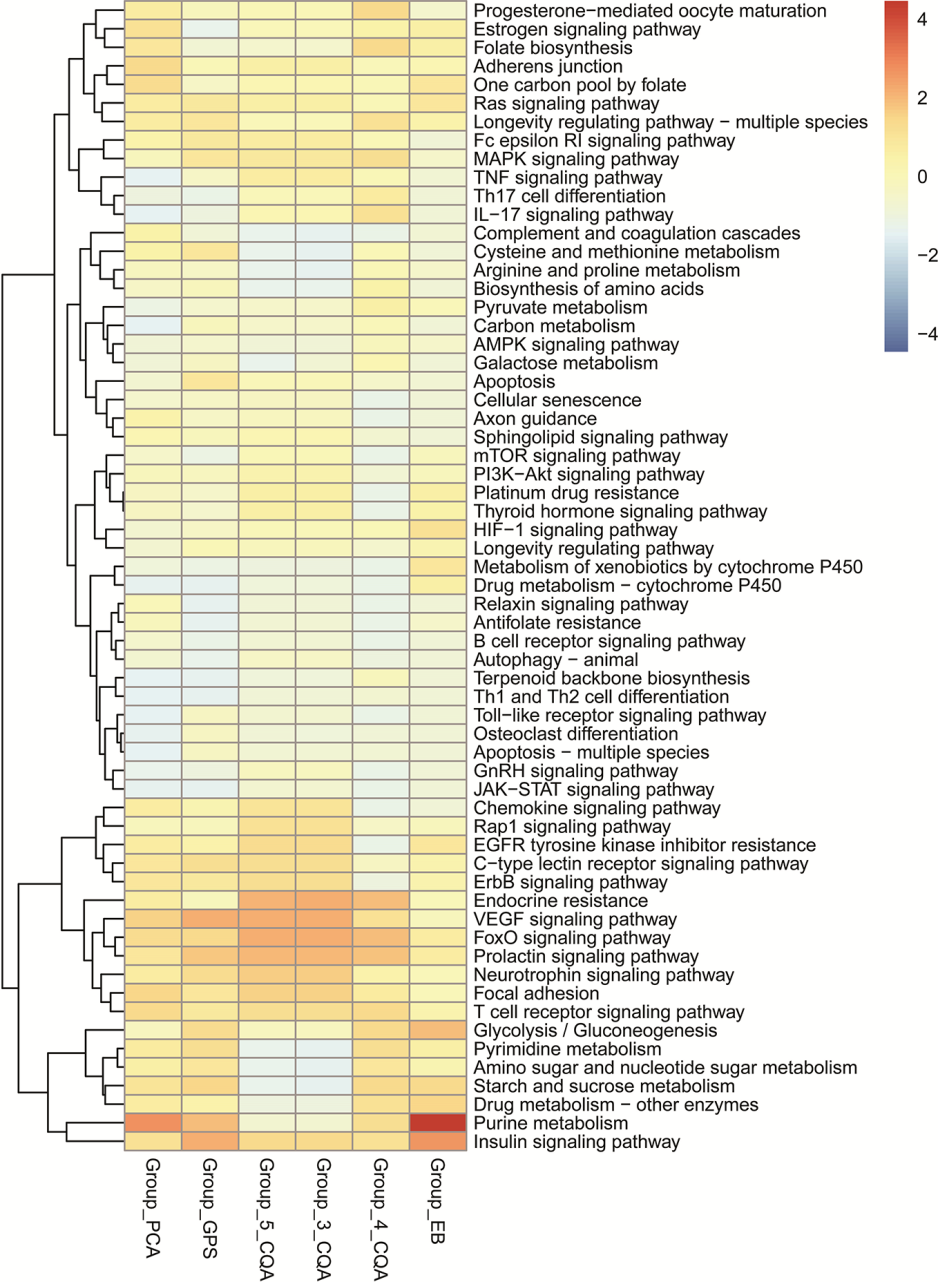
Heatmap of KEGG pathway enrichment analysis for the six main ingredients, 4-CQA, gentiopicroside (GPS), 5-CQA, protocatechuic acid (PCA), eleutheroside B (EB), and 3-CQA in *Acanthopanax senticosus* extract preparation (ASEP).

The KEGG enrichment analysis (**Figure 6**) showed that 4-CQA, GPS, 5-CQA, PCA, EB, and 3-CQA could mainly affect MAPK, Insulin, VEGF, FoxO, Purine metabolism, or Focal adhesion Pathways *in vivo*. Meanwhile, the heatmap of KEGG pathway enrichment analysis (**Figure 7**) further showed that the six studied ingredients could mainly interact with Insulin, FoxO and VEGF signaling pathways. And especially, all the six ingredients exhibited high-degree of similar color distribution in the pathways clustering, suggesting these ingredients could have a large extent to exert their cardiovascular effects *via* similar mulit-targets and mulit-pathways mechanisms. It also possibly exists cross-talk among the identified Insulin, FoxO, and VEGF signaling pathways for the interaction of the 6 studied ingredients *via* the shared target proteins (**Supplementary Figure S1**). Meanwhile, it must be mentioned that one ingredient binding to multiple targets may further exert the overall effectiveness on organisms, thereby promoting therapeutic effects, and more likely, multiple active ingredients may compete with each other and reduce the effectiveness.

A lot of studies have demonstrated that insulin signaling is essential for normal cardiovascular function, and lack of it (i.e., insulin resistance) will result in cardiovascular dysfunction and disease (Yu et al., 2011; Ormazabal et al., 2018). Researchers have concluded that preventing insulin resistance could avoid approximately 42% of myocardial infarctions in the participants during a simulated follow up period of 60 years using the Archimedes model, and a population representative of young nondiabetic adults aged from 20 to 30 years (Eddy et al., 2009). *A. Senticosus* extract (Liu et al., 2005), as well as its main components, such as syringin (EB) (Kim et al., 2017) and chlorogenic acid (3-CQA) (Ma et al., 2015) could improve insulin resistance in fructose-rich chow-fed rats, high-fat diet-fed mice and rats, respectively. In addition, insulin initiated PI3K-Akt-eNOS survival signaling, with nitric oxide (NO) as an ‘end effector’ delivering cardioprotection in health and disease (especially in ischemic heart disease) (Yu et al., 2011). The proposed cytoprotective mechanisms include improvement of coronary perfusion and subsequent contractile function *via* a potent vasodilatory effect (Schulz et al., 2004), regulation of myocardial oxygen consumption and metabolism *via* mitochondrial respiration control (Heusch et al., 2000), and so on. In one randomized controlled trial, a total of 160 cases with coronary heart disease and angina pectoris were randomly divided into two groups, observation group that received ASEP (60 ml/250 ml of 5% glucose injection, iv drip, qd) combined with standard medication (n = 80) and control group with standard medication alone (n = 80) for 14 days. The level of serum NO and NOS in observation group increased significantly compared with the control group (*P* < 0.05), suggesting that ASEP could effectively improve the vascular dysfunction status, so as to relieve the symptoms of angina (Lei, 2015).

Forkhead box O (FOXO) transcription factors are negatively regulated by the canonical insulin signaling pathway through PI3K and AKT, and regulates diverse gene expression programmes and affects many cellular processes, including cell cycle regulation, cell survival and metabolism (Eijkelenboom and Burgering, 2013), which could impact a number of clinical conditions such as CVDs, and protection against CVDs is one of FoxOs functions (Wang et al., 2014). Researchers demonstrated a remarkable atheroprotective effect of FoxO inhibition in mouse endothelial cells, and identified endothelial FoxO as a potential target for atherosclerosis prevention and treatment (Tsuchiya et al., 2012). Besides, the deletion of FoxO1 in the heart of high-fat diet mice prevented heart failure (Battiprolu et al., 2012). And GPS could activate the phosphorylation of AKT which down-regulates transcriptional activity of FoxO1 (Yang et al., 2018).

The recognition that VEGF signaling pathways are critical in physiological angiogenesis has led to the concept that these pathways are action targets for therapeutic angiogenesis, and the role of VEGF in the pathogenesis of CVDs such as atherosclerosis, ischemic heart disease, pulmonary hypertension, and vascular restenosis is of significance (Rajalakshmi et al., 2008). An increasing number of reports has supported the notion that growth factors and particularly VEGF contribute to atherosclerosis and can drive increases in rates of atherogenesis, and a substantial number of reported evidence has demonstrated that some dietary polyphenols could potently inhibit VEGF signaling, which is the major driver of angiogenesis, and results in reduced atherosclerosis and CVDs risk (Edwards and Kroon, 2014). For example, 3-CQA and PCA showed significant inhibitory effects on VEGF-induced angiogenesis in human umbilical vein endothelial cells and in the zebrafish model through down-regulating VEGFR2-mediated signaling transduction pathway and suppressing the expression of downstream angiogenesis-regulation genes (Lin et al., 2017; Hu et al., 2018).

Therefore, Insulin, FoxO and VEGF signaling pathways could be action target pathways of ASEP for CVDs therapies.

### Integrated PK Study of Multiple Ingredients of ASEP

**Figure 8:**
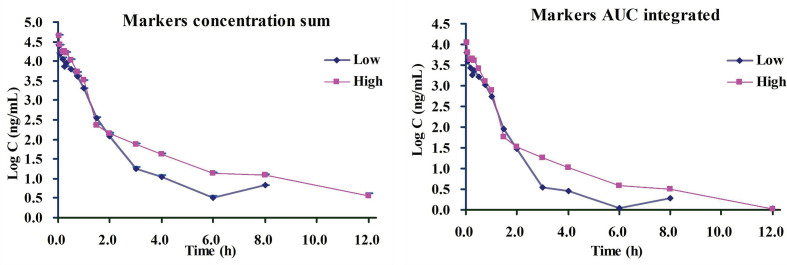
The drug plasma concentration-time curves by two integrated methods for *i.v.* administration of *Acanthopanax senticosus* extract preparation (ASEP) (low and high dosages) (Mean ± SD, *n*=3).

Based on the above-finished systems pharmacology and bioinformatics investigations, we inferred that all the six ingredients including 4-CQA, GPS, 5-CQA, PCA, EB, and 3-CQA could be the important therapeutic ingredients to CVDs, and they could share most targets and pathways to exert their therapeutic effects, possibly due to their similar molecular structures. Besides, the ordering of the contents of these components in ASEP were obviously higher than others (Xie et al., 2019), and the multiple-component PK studies showed their favorable PK properties (e.g., detectable in plasma). Accordingly, considering all these factors, the six main ingredients, 4-CQA, GPS, 5-CQA, PCA, EB, and 3-CQA should be selected as drug markers to profile the *in vivo* behavior of ASEP. All these provide supportings to characterize the whole *in vivo* process of ASEP by integrating the plasma drug concentration of these ingredients using simple methods, like “plasma drug concentration sum method” and the “AUC weighting integrated method” (Yao et al., 2017; Shi et al., 2018; Yao et al., 2018; Huang et al., 2019). Therefore, we next calculated the total concentrations of the markers in rat plasma according to the previous reports. The integrated plasma drug concentration–time curves are shown in **Figure 8**. The PK parameters listed in **Table 1** indicate no significant differences for almost all the PK data between the plasma drug concentration sum method and AUC weighting integrated method except for AUC_0–t_ and AUC_0–∞_. The AUC_0–t_ and AUC_0–∞_ values for the plasma drug concentration sum method were almost four times higher than those from the AUC weighting integrated method. The plasma drug concentration sum method reflected the whole influence of the concentration of each ingredient at each time point, while the AUC weighting integrated method highlighted the influence of the ingredients with larger exposure levels, such as EB and 5-CQA, on the overall exposure of ASEP.

As we know, formulating the dosage regimens is mainly according to PK parameters in clinic. However, in the classical PK studies on TCMs, the PK parameters of one or several individual components could be difficult to formulate the dosage regimens, because different individual components could have different PK parameters, and it is not reasonable to use one or several PK markers to represent the whole PK properties of TCMs. Thus, this integrated PK study could provide method reference for the clinical PK investigation of ASEP, and one set of PK parameters obtained from the integrated PK study of ASEP could be beneficial to the formulation of clinical rational dosage regimens.

## Conclusion

In this paper, an integrated PK of ASEP in rats was explored by combination of multi-component PK, systems pharmacology and bioinformatics analysis. The PK study of multiple ingredients from ASEP in rats revealed that the six ingredients, 4-CQA, GPS, 5-CQA, PCA, EB, and 3-CQA owned favorable PK properties. Further, by the network pharmacology, molecular docking and DO analysis, and KEGG pathway enrichment analysis, we inferred that the six ingredients could be the main active substances and share common multiple targets and multiple pathways against CVDs. Based on these, the six ingredients were subsequently selected as PK markers for characterizing the *in vivo* process of ASEP. Finally, the integrated PK study was carried out by integrating the plasma drug concentration of the selected PK markers with plasma drug concentration sum method and the AUC weighting integrated method. Our study provides novel insights into the therapeutic effects and mechanisms of ASEP against CVDs, and provides insights into the integrated PK investigation on anti-CVDs TCM injections.

Generally, also the present results suggested these ingredients could have a large extent to exert their cardiovascular effects *via* similar mulit-targets and mulit-pathways, which encouraged us to procedure the integrated PK study by the simple plasma-drug concentration sum or plasma-drug AUC weighted methods. In one word, the virtual screening, network pharmacology and molecular docking study could provide auxiliary evidences for the integrated PK study, and could make a small step forward for us to conquer the big problem, how to characterize the whole *in vivo* process of a TCM. In the future, we should focus on exploring the feasible and simple method to experimentally disclose the effect targets and pathways for complex TCM system.

## Data Availability Statement

The raw data supporting the conclusions of this article will be made available by the authors, without undue reservation.

## Ethics Statement

The animal study was reviewed and approved by the Animal Care and Use Committee of the College of Animal Science (College of Bee Science), Fujian Agriculture and Forestry University (Approval Number: PZCASFAFU2019004).

## Author Contributions

PS conceived, designed, analyzed the data, and wrote the paper. YX, RX, and ZL performed the experiments and analyzed the data. HY conceived, designed, analyzed the data, and revised the paper. SW conceived, designed, and revised the paper.

## Funding

The authors gratefully acknowledge the financial supports of the National Nature Science Foundation of China (81973558), the Natural Science Foundation of Fujian Province (2018J01596), the Distinguished Young Scientific Research Talents Plan in Universities of Fujian Province (2017), the Program for New Century Excellent Talents in Fujian Province University (2018), and the Joint Funds for the Innovation of Science and Technology, Fujian province (2017Y9123).

## Conflict of Interest

The authors declare that the research was conducted in the absence of any commercial or financial relationships that could be construed as a potential conflict of interest.
