# Supplementary material for: An Integrated Pharmacokinetic Study of an Acanthopanax senticosus Extract Preparation by Combination of Virtual Screening, Systems Pharmacology, and Multi-Component Pharmacokinetics in Rats
Source: Front Pharmacol. 2020 Aug 14;11:1295. doi: 10.3389/fphar.2020.01295 (PMC7457137; doi:10.3389/fphar.2020.01295)
Supplement: Supplementary file 1 [file DataSheet_1.doc]

Supplementary Material

# An integrated pharmacokinetic study of an *Acanthopanax senticosus* extract preparation by combination of virtual screening, systems pharmacology and multi-component pharmacokinetics in rats

Peiying Shi^1,2†^, Yunjiao Xie^3†^, Rongfang Xie^3^, Zuan Lin^3^, Hong Yao^3*^, Shuang Wu^2*^

^1^ Department of Traditional Chinese Medicine Resource and Bee Products, College of Animal Science (College of Bee Science), Fujian Agriculture and Forestry University, Fuzhou, 350002, China

^2^ College of Horticulture, FAFU-UCR Joint Center and Fujian Provincial Key Laboratory of Haixia Applied Plant Systems Biology, Fujian Agriculture and Forestry University, Fuzhou, 350002, China

^3^ Department of Pharmaceutical Analysis, School of Pharmacy, Fujian Medical University, Fuzhou 350122, China

*** Correspondence:**Hong Yao
hongyao@mail.fjmu.edu.cn or yauhung@126.com Shuang Wu
wus@fafu.edu.cn

^†^ These authors have contributed equally to this work.

**1 Supplementary Figures**

**
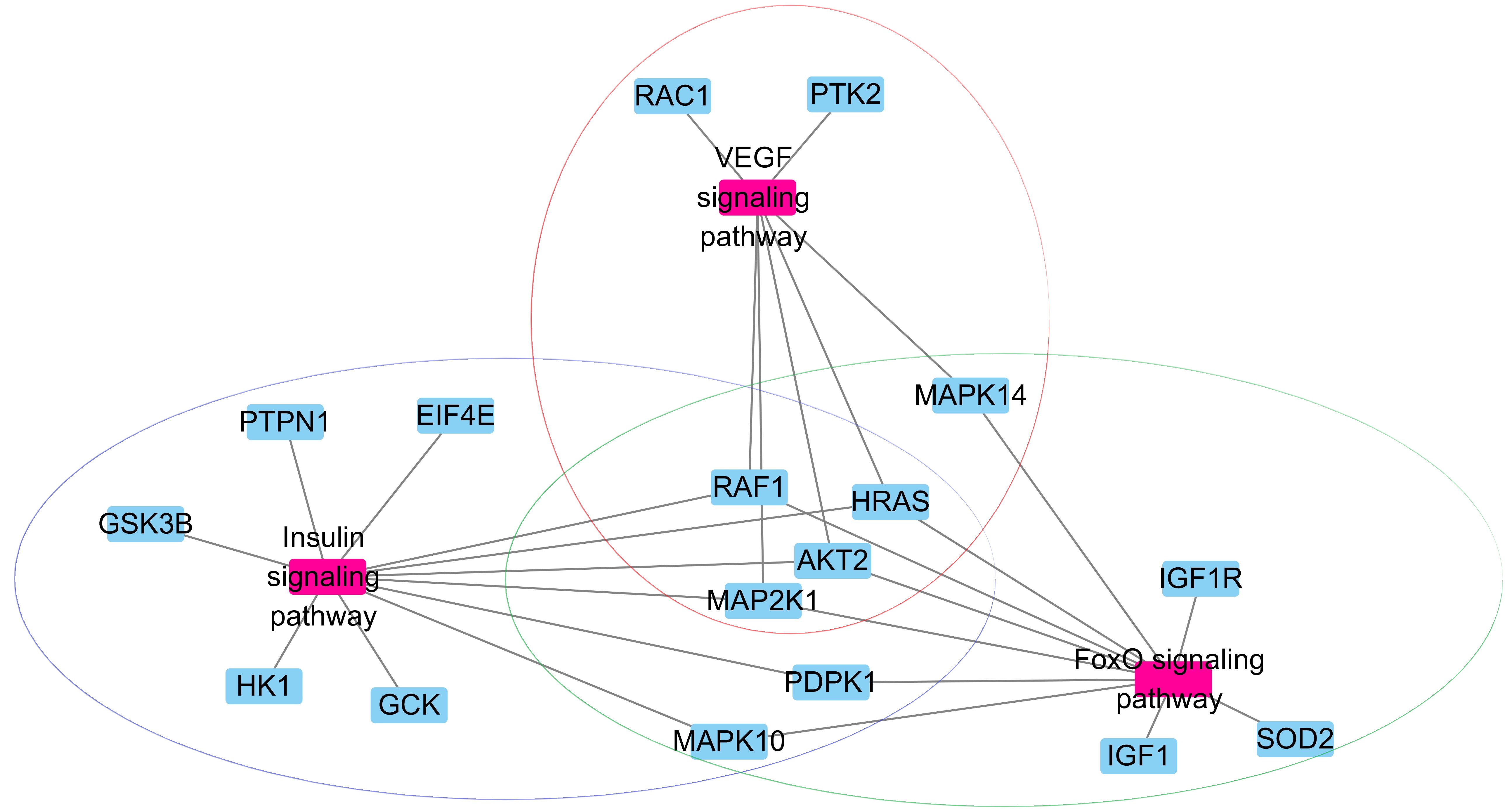
**

**Supplementary Figure S1** Target-Pathway (T-P) networks and their cross-talk relationship via the shared target proteins (like RAF1, HRAS, AKT2, MAP2K1, etc.) for the 6 studied ingredients.

**2 Supplementary Tables**

**Supplementary Table S1 The regression equation, linear range, r^2^, LOD and LOQ of 6 compounds in the ASEP.**

| **Compound** | **Regression equation** | **Linear range（ng/mL）** | **r^2^** | **LOD** | **LOQ** |
| --- | --- | --- | --- | --- | --- |
| 5-CQA | y = 0.42x + 0.0069 | 10-20000 | 0.992 | 2.5 | 5.0 |
| PCA | y = 0.24x + 0.0072 | 2-10000 | 0.990 | 0.5 | 1.0 |
| 3-CQA | y = 0.31x + 0.0056 | 2-15000 | 0.996 | 1.0 | 2.0 |
| EB | y = 0.26x + 0.016 | 5-10000 | 0.993 | 2.5 | 5.0 |
| 4-CQA | y = 0.34x + 0.0061 | 5-20000 | 0.991 | 2.5 | 5.0 |
| GPS | y = 0.86x + 0.053 | 10-12500 | 0.996 | 2.0 | 6.0 |

**Supplementary Table S2 The precision and accuracy of 6 compounds of ASEP in rat plasma.**

| **Analyte** | **Concentration (ng/mL)** | **Intra-day (n=5)** | | | **Inter-day (n=5)** | | |
| --- | --- | --- | --- | --- | --- | --- | --- |
|  |  |  |  |  |  |  |  |
|  |  | **Detected** | **RSD** | **Accuracy** | **Detected** | **RSD** | **Accuracy** |
|  |  | **Mean±SD (ng/mL)** | **(%)** | **(RE, %)** | **Mean±SD**  **(ng/mL)** | **(%)** | **(RE, %)** |
| 5-CQA | 30 | 28.71±0.76 | 3.49 | -4.30 | 28.74±1.43 | 7.06 | -4.20 |
|  | 1600 | 1763.64±43.86 | 3.70 | 10.23 | 1794.19±56.91 | 4.19 | 12.14 |
|  | 16000 | 14369.99±165.32 | 1.41 | -10.19 | 14639.53±318.14 | 2.69 | -8.50 |
| PCA | 5 | 4.76±0.53 | 14.94 | -4.80 | 4.68±0.36 | 10.17 | -6.40 |
|  | 400 | 436.30±16.57 | 4.53 | 9.08 | 428.89±26.40 | 7.72 | 7.22 |
|  | 8000 | 7646.32±111.36 | 1.75 | -4.42 | 7707.97±131.43 | 2.05 | -3.65 |
| 3-CQA | 5 | 5.61±0.10 | 2.70 | 12.20 | 5.27±0.60 | 14.50 | 5.40 |
|  | 600 | 587.87±13.83 | 2.70 | -2.02 | 589.27±46.07 | 8.97 | -1.79 |
|  | 12000 | 11563.83±233.60 | 2.50 | -3.63 | 11722.27±252.85 | 2.68 | -2.31 |
| EB | 15 | 15.41±0.65 | 4.94 | 2.73 | 16.08±0.87 | 6.92 | 7.20 |
|  | 800 | 777.32±17.71 | 2.79 | -2.83 | 783.06±47.38 | 7.82 | -2.12 |
|  | 8000 | 8914.78±311.66 | 4.39 | 11.43 | 8801.70±424.79 | 6.20 | 10.02 |
| 4-CQA | 10 | 11.72±0.81 | 8.98 | 17.20 | 10.43±1.25 | 14.24 | 4.30 |
|  | 1000 | 980.80±21.40 | 3.03 | -1.92 | 985.20±53.87 | 6.67 | -1.48 |
|  | 16000 | 15490.54±285.27 | 2.32 | -3.18 | 16356.96±828.83 | 5.75 | 2.23 |
| GPS | 20 | 19.93±1.29 | 7.80 | -0.35 | 19.55±1.55 | 8.86 | -2.25 |
|  | 1250 | 1366.22±46.60 | 3.99 | 9.30 | 1309.25±56.86 | 5.49 | 4.74 |
|  | 10000 | 9943.43±275.07 | 3.80 | -0.57 | 10032.43±423.74 | 5.12 | 0.32 |

**Supplementary Table S3 The extraction recoveries and matrix effect of 6 compounds of ASEP in rat plasma.**

| **Analyte** | **Concentration (ng/mL)** | **Extraction recovery (n=5)** | | **Matrix effect (n=5)** | |
| --- | --- | --- | --- | --- | --- |
|  |  |  |  |  |  |
|  |  | **Mean± SD (%)** | **RSD (%)** | **Mean±SD (%)** | **RSD (%)** |
|  |  |  |  |  |  |
| 5-CQA | 30 | 104.24±2.76 | 4.20 | 94.15±2.88 | 8.01 |
|  | 1600 | 101.77±2.53 | 4.11 | 92.04±3.01 | 4.96 |
|  | 16000 | 86.08±0.99 | 3.72 | 94.04±2.65 | 2.07 |
| PCA | 5 | 95.99±10.69 | 6.66 | 128.61±6.55 | 8.49 |
|  | 400 | 102.58±3.90 | 6.85 | 89.41±4.75 | 2.28 |
|  | 8000 | 92.45±1.35 | 2.40 | 81.65±1.36 | 4.82 |
| 3-CQA | 5 | 109.21±1.95 | 11.78 | 94.16±7.19 | 7.16 |
|  | 600 | 89.48±2.11 | 5.05 | 96.82±3.75 | 4.28 |
|  | 12000 | 89.55±1.81 | 2.84 | 91.86±1.63 | 2.72 |
| EB | 15 | 101.12±4.27 | 2.85 | 123.32±2.63 | 4.58 |
|  | 800 | 90.83±2.07 | 3.49 | 80.62±2.1 | 3.35 |
|  | 8000 | 95.45±3.34 | 6.64 | 72.08±3.97 | 3.54 |
| 4-CQA | 10 | 113.55±7.85 | 9.33 | 107.09±7.73 | 9.92 |
|  | 1000 | 81.51±1.78 | 2.92 | 95.17±1.91 | 2.41 |
|  | 16000 | 90.59±1.67 | 4.70 | 86.35±2.97 | 4.48 |
| GPS | 20 | 81.7±5.29 | 12.47 | 110.79±11.87 | 5.44 |
|  | 1250 | 93.41±3.19 | 3.66 | 93.27±2.5 | 3.97 |
|  | 10000 | 92.76±2.57 | 6.99 | 94.11±5.63 | 1.43 |

**Supplementary Table S4 The stability of 6 compounds of ASEP in rat plasma.**

| **Analyte** | **Spiked (ng/mL)** | **6h at room  temperature** | | **10h in  auto-sampler  vials** | | **Freeze-thaw  stability (three cycles)** | | **Long-term  stability (-80**℃**,15 days)** | |
| --- | --- | --- | --- | --- | --- | --- | --- | --- | --- |
|  |  |  |  |  |  |  |  |  |  |
|  |  |  |  |  |  |  |  |  |  |
|  |  | **RE** | **RSD** | **RE** | **RSD** | **RE** | **RSD** | **RE** | **RSD** |
|  |  | **(%)** | **(%)** | **(%)** | **(%)** | **(%)** | **(%)** | **(%)** | **(%)** |
| 5-CQA | 30 | -3.10 | 5.36 | -11.38 | 9.10 | 9.22 | 7.59 | 12.43 | 3.41 |
|  | 1600 | -5.55 | 2.72 | -9.33 | 1.65 | 3.99 | 4.38 | 5.71 | 4.10 |
|  | 16000 | -0.66 | 2.49 | -8.59 | 2.23 | -8.36 | 3.71 | -4.85 | 3.17 |
| PCA | 5 | -6.60 | 0.72 | -2.62 | 2.07 | -0.31 | 4.61 | -9.72 | 5.45 |
|  | 400 | -4.01 | 1.96 | -6.89 | 3.44 | 0.85 | 4.57 | -3.16 | 6.83 |
|  | 8000 | 0.16 | 2.06 | -5.63 | 2.16 | -0.14 | 2.82 | -10.60 | 2.65 |
| 3-CQA | 5 | -8.18 | 9.85 | -10.45 | 3.03 | 8.84 | 6.13 | 3.64 | 3.12 |
|  | 600 | -2.06 | 0.82 | -4.50 | 3.27 | -8.13 | 3.10 | 10.42 | 6.08 |
|  | 12000 | -6.66 | 3.66 | -7.73 | 2.74 | -4.27 | 2.80 | -1.46 | 2.12 |
| EB | 15 | -5.59 | 6.88 | 12.12 | 4.24 | 4.10 | 5.29 | -8.20 | 7.62 |
|  | 800 | -14.91 | 4.01 | -0.97 | 5.14 | -0.75 | 1.85 | -4.54 | 1.56 |
|  | 8000 | -4.48 | 2.51 | 1.92 | 5.08 | -4.14 | 2.86 | 6.11 | 2.50 |
| 4-CQA | 10 | -6.42 | 12.45 | -16.89 | 9.40 | 19.90 | 9.63 | 17.58 | 2.60 |
|  | 1000 | 9.60 | 6.56 | 14.39 | 2.73 | -0.64 | 5.44 | 14.43 | 2.06 |
|  | 16000 | -7.63 | 2.73 | -6.85 | 4.20 | -3.17 | 3.35 | -1.64 | 2.21 |
| GPS | 20 | -10.12 | 3.18 | 10.09 | 8.16 | 14.78 | 3.63 | 12.72 | 3.84 |
|  | 1250 | -6.72 | 5.88 | 2.52 | 6.41 | 1.22 | 6.53 | 4.29 | 3.18 |
|  | 10000 | -13.56 | 8.56 | -9.29 | 1.25 | -2.10 | 2.86 | 9.68 | 3.96 |

**Supplementary Table S5** Total score results of molecular docking studies of 6 compounds in the active sites of 14 proteins performed using SYBYL-X 1.3.

| Proteins (PDB ID) | 4-CQA | 5-CQA | 3-CQA | GPS | EB | PCA |
| --- | --- | --- | --- | --- | --- | --- |
| GSK3B (1q4l) | 8.0718 | 7.8575 | 8.2804 | 7.2261 | 6.2515 | 3.9519 |
| PDPK1 (2pe2) | 6.6991 | 7.8468 | 7.1602 | 5.8393 | 4.4464 | 3.6851 |
| SOD2 (1xdc) | 8.5465 | 8.0681 | 7.2327 | 6.6780 | 5.9545 | 4.7653 |
| PAH (1dmw) | 5.2412 | 5.0825 | 4.3771 | 4.6638 | 4.1770 | 2.2015 |
| NOS2 (2nsi) | 7.6099 | 6.4161 | 6.6623 | 5.6900 | 4.6715 | 3.8535 |
| XIAP (3cm2) | 7.1040 | 8.5371 | 7.9309 | 5.7108 | 5.8358 | 4.9286 |
| SHMT1 (1bj4) | 6.1473 | 7.2677 | 7.5340 | 5.0747 | 3.8787 | 2.3781 |
| RHOA (1kmq) | 10.4785 | 9.1391 | 10.1918 | 5.8394 | 5.3507 | 4.9547 |
| PLAU (1f5l) | 5.0299 | 5.1781 | 5.6502 | 4.2389 | 4.2402 | 1.9715 |
| MTHFD1 (1dia) | 8.9799 | 10.2459 | 9.9294 | 6.8720 | 5.9755 | 4.8693 |
| IL2 (1qvn) | 6.6847 | 6.5839 | 7.3659 | 5.9082 | 5.6980 | 3.4630 |
| GP1BA (1p9a) | 5.7032 | 5.8391 | 5.8629 | 3.7582 | 2.1713 | 3.3746 |
| CRAT (1s5o) | 7.6577 | 6.4813 | 7.0994 | 5.3788 | 4.8448 | 3.8996 |
| BST1 (1isj) | 6.9709 | 6.1645 | 6.5463 | 4.7102 | 4.5286 | 3.1816 |

**Supplementary Table S6** Binding free energy results of molecular docking studies of 6 compounds and 14 proteins performed using LeDock (kcal/mol).

| Proteins (PDB ID) | 4-CQA | 5-CQA | 3-CQA | GPS | EB | PCA |
| --- | --- | --- | --- | --- | --- | --- |
| GSK3B (1q4l) | -5.67 | -5.94 | -5.80 | -5.45 | -5.74 | -3.10 |
| PDPK1 (2pe2) | -5.45 | -5.41 | -5.49 | -5.87 | -5.51 | -3.23 |
| SOD2 (1xdc) | -3.31 | -3.43 | -3.48 | -2.86 | -2.81 | -2.35 |
| PAH (1dmw) | -4.55 | -4.93 | -4.88 | -4.92 | -4.84 | -3.07 |
| NOS2 (2nsi) | -5.27 | -6.07 | -6.70 | -5.85 | -5.22 | -3.57 |
| XIAP (3cm2) | -5.38 | -5.63 | -5.42 | -5.14 | -5.70 | -2.81 |
| SHMT1 (1bj4) | -4.65 | -4.80 | -4.93 | -4.55 | -4.47 | -3.01 |
| RHOA (1kmq) | -6.43 | -6.53 | -6.96 | -6.79 | -6.41 | -4.33 |
| PLAU (1f5l) | -5.45 | -5.52 | -5.32 | -5.13 | -5.15 | -3.48 |
| MTHFD1 (1dia) | -5.33 | -5.26 | -5.12 | -5.01 | -5.11 | -2.97 |
| IL2 (1qvn) | -5.90 | -5.90 | -5.75 | -5.76 | -5.49 | -3.31 |
| GP1BA (1p9a) | -3.27 | -3.41 | -3.35 | -3.01 | -3.16 | -2.04 |
| CRAT (1s5o) | -5.51 | -5.72 | -5.76 | -5.44 | -5.01 | -3.22 |
| BST1 (1isj) | -5.28 | -5.08 | -5.58 | -5.37 | -4.70 | -3.50 |
